# Psychological Inquiries

**Published:** 1854-10-01

**Authors:** 


### THE JOURNAL

OF

### PSYCHOLOGICAL MEDICINE

AND

### MENTAL PATHOLOGY.

OCTOBER 1, 1854.

### Art. I.—PSYCHOLOGICAL INQUIRIES.

So long as metaphysicians limit their inquiries to the laws of mental action, and omit therefrom an investigation of the organic conditions under which that action takes place, so long will mental philosophy, and the whole theory of human nature in all its aspects, be incomplete. The power of the soul over the body has been amply illustrated, because little physiology and less anatomy are requisite for the inquiry; the power of the body over the soul, on the contrary, has been left in obscurity, because a profound physiology, a correct anatomy, and a large experience of human nature in its ordinary aspects, are necessary to its elucidation. The speculative philosopher, withdrawn from the world, and abstracted in the contemplation of his own consciousness, knows little of these requisites; the observant, experienced, and scientific medical practitioner knows much. Such an one (as may be deduced from internal evidence) is the author of the charming little volume before us,* and such appears to be the opinion he has formed. He remarks:—

"It is the business of medical practitioners to study not only the influence of the mind on the body, but also that of the body on the

* Psychological Inquiries: in a Series of Essays, intended to illustrate the Mutual Relations of the Physical Organization and the Mental Faculties. London, 1854. 12mo, pp. 264.

mind; and in so doing, they have the opportunity of learning, more than others, to trace moral effects to physical causes. Where others complain of a fretful and peevish temper, it may be that they are led to make allowance for the difficulty of self-restraint where there is a superabundance of lithic acid in the blood, or an organic disease of the viscera. In the catalepsy induced in a nervous girl by the so-called mesmeric passes, they see only one of the numerous phases of that multiform disease, hysteria; and in the mischievous, and sometimes even in the benevolent enthusiast, who by his sincerity and earnestness enlists in the cause which he undertakes the sympathy of the multitude, their more experienced observation will often detect the commencement of illusions and the germ of insanity."

Not that the shrewd common-sense of mankind has not readily detected many relations between the corporeal condition and the mental state, and advises you, if you would secure a favour from your patron, to ask it after dinner, and not before. Practical views are not overlooked. The midnight debauch is equally as potent in its influences on the soul as fasting.

> "—— Quin corpus onustum

> Hesternis vitiis animum quoque prægravat unà,

> Atque affigit humo divinæ particulum auræ."

"It would, however, be a very great mistake to regard this kind of knowledge as being altogether peculiar to medical practitioners. In fact, the connexion between the mind and body is in many instances too palpable to be overlooked by any practical observer of mankind. For example, it is referred to by Lord Chesterfield, when he says that many a battle has been lost because the general had a fit of indigestion; and you may recollect that I stated on a former occasion that Mr. Chadwick had already exposed the operation of living in an unwholesome atmosphere as inducing the habit of gin-drinking, with all the frightful moral consequences which follow in its train. Still, it must be admitted that members of the medical profession have better opportunities of obtaining knowledge of this kind than most other persons; and hence it is that in many things which, even in these days of education, and in spite of the advancement of knowledge, others regard with wonder, as the result of some unknown and mysterious agency, they, with some rare exceptions, see nothing that is not to be explained on well-known principles, or in any degree more remarkable than the exploits of M. Robin or other conjurors."

Practitioners have attempted from time to time to *popularize* their knowledge on these important psychological subjects, hoping that that knowledge would constitute an effectual antidote to the belief in the follies and knaveries incidentally referred to in the paragraph just quoted. Unfortunately, the mode adopted has proved little to the taste of these credulous people, whose most agreeable mental aliment is excitement; those only with such mental endowments as restrain them from a wild credulity, appreciate the depth and force of the arguments advanced.

In the present volume we have an attempt of this kind undertaken with skill and taste, and, therefore, more probably successful. Less classical and technical, and less profound than Sir Henry Holland's kindred and admirable work, "Chapters on Mental Physiology," it is perhaps the more readable by the non-professional but intelligent public.

The book is written in the form of a dialogue between a London surgeon of large experience, named *Ergates* (a worker), a member of the legal profession, *Crites* (a judge), and an enlightened politician—a retired right honourable, designated *Eubulus*. The two latter advance such objections to the physiological and psychological views of Ergates, or adduce such corroborations as would naturally arise in the minds of men of cultivated intellect and large experience of mankind, but without that special information which the observant and scientific practitioner possesses in virtue of his profession and his office.

The opening of the first dialogue introduces us to the *personæ* of the conversation. Ergates is the speaker.

"The Session of Parliament was drawing to a close. Ministers took advantage of the approach of the grouse-shooting season to hurry through the two Houses the various Bills which they could not venture to postpone for another year. Some official and professional persons still lingered in the clubs, but the houses in the squares were deserted, and there was an end for the season of what is called κατ' ἐξοχήν,—London society. Meeting accidentally a friend, whom I shall distinguish by the name of Crites, I expressed my surprise at seeing him still in London. 'Our court,' said he, 'has been sitting later than usual, but I am now emancipated, and I am about to pay a long-promised visit to our friend Eubulus. I know that it would afford him the greatest pleasure if you would accompany me as his visitor.'

"Eubulus had been my intimate friend in early life. As boys, we had wandered together through our native woods; as young men, we had similar pursuits and tastes, had admired the same poetry, and had speculated together on subjects beyond the reach of human wit; but afterwards, being engaged in different professions, and our roads in life lying in different directions, we had parted company, and, as we travelled onwards, had only occasional glimpses of each other. Still, whenever we met, the influence of old associations remained unimpaired; we were as intimate as formerly, and seemed to know more of each other than of any friend whom we had acquired at a later period of life.

"It was two or three years before the period of which I am now speaking that Eubulus, finding that his health was scarcely equal to the duties of the office which he held, and that between what he had obtained by inheritance and a retiring pension he had sufficient fortune to meet the reasonable demands of himself and his family, had gone to reside on a property which he possessed at the distance of a hundred miles from the metropolis; and here he had repeatedly urged me to be his guest. Nothing could be more agreeable to me than the proposal which Crites made, and the result was, that in less than forty-eight hours we were both seated in a carriage on the railway, and, in the course of a few hours more, were set down within a mile of our destination."

We have given this quotation at length, to illustrate the natural and unaffected style of the author. The house and landscape are then described, and the feelings of the old friends or schoolfellows on renewing their social intercourse, are analyzed. We cannot refuse our readers the pleasure of perusing the following passages:—

"It seemed at times as if we had gone back to the period of our early life. We expressed ourselves as freely as when we were young, having before us the unknown country which we were about to explore. Still we were sensible that we were not what we had been formerly. The world was no longer that fairyland which our imagination was wont to furnish with its own images. We knew it, and the people in it, and we knew ourselves, better than when we began our journey. We had lost the joys of hope and expectation, but we had lost also many of the anxieties which not unfrequently obscured our brighter visions, and years had not rolled over us without leaving us, in the realities of life, many worthy subjects of contemplation."

The friends thus met became for the while peripatetic philosophers, and their host's domain and neighbourhood their "groves of Academe." They were each glad to leave the whirl of life and its wearying routine for the more congenial pursuits of philosophy. Such seem to be the nearest approach to the intellectual enjoyments of the future and better world, provided they are directed to the highest and holiest objects of investigation.

—— "What if earth

Be but the shadow of heaven, and things therein

Each to other like, more than on earth is thought?"

In giving a critical analysis of this work, we shall deviate from the plan of our author, because the limits allotted to us are restricted. Our analysis must, in fact, end in a *synthesis*. And first we take our author's opinions as to mind and matter. The panoramic view of an extensive landscape in one of those days preceding rain, when the atmosphere is unusually transparent, raises the imagination to high thought.

"'I never,' said Eubulus, 'find myself left to my own contemplations in a situation such as this, without a feeling of wonder at myself and my own existence. Here am I,—I mean I who feel and think,—pent up within the narrow dwelling of my own body, yet taking cognizance of things remote in space, not only of those which belong to our own world, but of those in the vast universe around us. Marvellous as this may be, let us wait but for a few hours, and we have what is still more marvellous. By the aid of a tube and a few glasses, I may become acquainted with other objects, suns, and worlds, distant from us not only in space, but also in time, which I see not as they now are, but as they were many thousands of years before I myself was in existence. I do not say that such reflections prove more than may be proved in other ways, but they certainly impress my mind more strongly with the conviction that, as a percipient, conscious, and intelligent being, I belong to a mode of existence wholly different from that of the senseless bodies by which I am surrounded, and that (even independently of the evidence afforded by revelation) there is nothing unreasonable in the universal expectation of mankind (so universal, indeed, as to have almost the character of an instinct) that there is something in us which will remain, and be capable of perception and thought—and it may be of pure and high aspirations—when the gross material fabric with which it is now associated has become resolved into its original elements.' "

To the argument of the materialist, that we know nothing of mind except through organization, it is answered, the existence of one's own mind is the only thing of which we have indubitable knowledge; it is, in fact, as much a contradiction to doubt the existence of one's own own mind as that two and two make four. Then there is the evidence in favour of something distinct from "senseless" matter presented by the phenomena of creation, in which from the grandest to the smallest, from those presented in boundless space by vast orbs in relation to each other, to those presented by microscopic cells and nuclei—also in relation to each other—there is one ever active, ever constant something present, by virtue of which something the phenomena are all guided in orderly sequence to an object as surely as the masses of senseless matter are regulated by the force of gravity. It is as certainly demonstrable that there is a designing and intellectually regulating force, as that there is a centrifugal and a centripetal force. Our author states that the evidence of intention and design is more especially manifested in the vegetable and animal creations. In this we differ from him: it is certainly more obvious, but it is as fully manifested in the planetary movements, and other Cosmic phenomena. In the twenty-first and twenty-second chapters of "Paley's Natural Theology,"* numerous illustrative facts are stated. In a note to chapter twenty-fifth† is also an illustration drawn from La Place. It is to the effect that the rising again of the sun on the morrow of any given day "is above two million times less probable than the truth of the position that the motions in our system were designed by one First Cause."

Mind and matter, then, do both exist. Of that proposition there can be no reasonable doubt; but inasmuch as there be they who do doubt, let us, for the sake of a starting-point, allowing that

* With Illustrative Notes by Lord Brougham and Sir Charles Bell. 1836. Vol. ii. p. 1.

† Ed. cit. p. 102.

matter only has existence, come to some understanding as to how we shall designate that something which constitutes our self-consciousness, and how we shall describe that which so operates as to carry on phenomena to the completion of a special and particular purpose; which purpose being attained, we are happy or pleased, or things continue in the same pre-arranged order; but which purpose *not* being accomplished, we are unhappy, or suffer pain, or things are in another order than that which is normal,—i.e., *dis*order. We ask the materialist how he would designate this designing, effectuating, something in creation, and this feeling, thinking, willing agent in ourselves? To call either a property of matter is to tell us nothing, for in no respect does it resemble any one of the *recognised* properties of matter. Turn the subject how we may, we come inevitably back to the common conclusion of mankind, and designate it as MIND.

Mind, then, is alike at work in creation, and in the wonderful vital mechanism termed Man. How far is the one form identical in its essence and modes of action with the other? As to vertebrate animals, the common nature and mode of action of mind is an acknowledged fact. It is true that from time to time specific differences are erroneously constituted into generic by those who endeavour to exalt man by separating him, whether considered in his mental or corporeal relations, from his fellow-creatures below him in intellectual development. This is, however, not in accordance with observed facts. The argument for the identity is well put by the author.

"ERGATES.—It may be, as I observed on a previous occasion, that some of those which are usually regarded as the very lowest form of animal life, have no endowments superior to those which belong to vegetables. Setting these aside, however, I apprehend that no one who considers the subject can doubt that the mental principle in animals is of the same essence as that of human beings; so that even in the humbler classes we may trace the rudiments of those faculties to which, in their state of more complete development, we are indebted for the grandest results of human genius. We cannot suppose the existence of mere sensation without supposing that there is something more. In the stupid carp which comes to a certain spot, at a certain hour, or on a certain signal, to be fed, we recognise at any rate the existence of memory and the association of ideas. But we recognise much more than this in the dog who assists the shepherd in collecting his sheep in the wilds of the Welsh mountains. Locke, and Dugald Stewart following him, do not allow that 'brute animals have the power of abstraction.' Now, taking it for granted that abstraction can mean nothing more than the power of comparing our conceptions with reference to certain points to the exclusion of others—as, for example, when we consider colour without reference to figure, or figure without reference to colour—then I do not see how we can deny the existence of this faculty in other animals, any more than in man himself. In this sense of the word, abstraction is a necessary part of the process of reasoning which Locke defines as being 'the perception of the agreement or disagreement of our ideas.' But who can doubt that a dog reasons while he is looking for his master, whom he has lost; or when he is seeking his way over an unknown country?"

It is added, that "the minds of the inferior animals are essentially of the same nature with that of the human race; and that of those various and ever-changing conditions of it, which we term the mental faculties, there are some of which we may not discover traces, more or less distinct, in other creatures." This is a fundamental principle of the highest importance in psychological researches, for, placed in another form, it is this, *that the nature of the human mind and its relation to organization may be investigated through the mental phenomena of the inferior animals.* If a psychologist, thoroughly imbued with the truth of this proposition, sees in all the acts of these, his lower fellow-creatures, the reflected image of the working of his own mind, he cannot watch the instinctive or other acts of the smallest or lowest, without feeling those touches of nature which make the whole world kin; or without obtaining wonderful glimpses into his own mental being, and thus day by day acquiring fresh knowledge. Nor will his observations and sympathies be limited to animals, for as the mind evolves the ideas which naturally flow from so suggestive a principle, it passes from one gradation of life to another, ever descending by imperceptible steps, until at last the ever-varied phenomena of vegetable life are brought into the same category, and the identity with his own of *mind* in *creation*, as well as in animal life, is made manifest.

——"By gradual scale sublimed,
The vital spirits aspire, to animal,
To intellectual; give both life and sense,
Fancy and understanding; whence the soul
Reason receives, and reason is her being,
Discoursive or intuitive."

The fact is, that no man is properly qualified to observe, compare, and estimate these mental phenomena in the organized beings below him, until he has thus descended from that lofty pedestal upon which his pride of place has exalted him. That pride hinders the operation of his powers, whether of observation or of reflection, by restricting them to the narrow sphere of his own life. His prejudices blind him, or pervert his judgment; they harden his heart by contracting his sympathies; and so the hidden chords of his nature, which are in unison with those of the creatures below, rarely vibrate to the awakening of new ideas, or vibrate but imperfectly. The vast field for that comparative psychology which can alone release us from the circle of metaphysical subtleties in which we have hitherto trod, is partly indicated in the following quotation:—

"If even a portion of the observations made by the younger Huber on ants be well founded, these little creatures must be regarded as possessing, in addition to their instincts, no small portion of intelligence. It is observed by a modern writer, that there is hardly a mechanical pursuit in which insects do not excel. They are excellent weavers, house-builders, architects. They make diving-bells, bore galleries, raise vaults, construct bridges. They line their houses with tapestry, clean them, ventilate them, and close them with admirably-fitted swing doors. They build and store warehouses, construct traps in the greatest variety, hunt skilfully, rob and plunder. They poison, sabre, and strangle their enemies. They have social laws, and common language, division of labour, and gradations of rank. They maintain armies, go to war, send out scouts, appoint sentinels, carry off prisoners, keep slaves, and tend domestic animals. In short, they are a miniature copy of man rather than of the inferior vertebrata.* Of these things, which have been thus graphically described, much may indeed be referred to the operation of instincts, or to what Dr. Carpenter terms 'unconscious cerebration:' but surely it involves a considerable *petitio principii* not to refer a part of them to a higher principle, bearing a resemblance, however remote, to human intelligence."

The attention of naturalists has been too exclusively directed to a few leading instincts in lower animals, such as the conservation of life, the union of the sexes, &c. But there are numerous emotions and passions displayed by them less obvious than these, but not less instructive. Of this kind is the following instance of a domestic tragedy, consequent on the hopes of "a son and heir," entertained by a pair of canaries, being utterly blasted. A lady of our acquaintance possessed the birds, and related to us the melancholy history. After the usual period of pairing, the hen bird commenced the process of incubation, and nothing could exceed the affectionate gallantry of her mate while thus engaged. His song was never wanting to cheer her, and in a variety of ways he showed the warm interest he took in her proceedings. At last the allotted period expired, and sad to relate the eggs were addled. So soon as the male found that no young birds appeared to share his affections with his mate, he fell upon her, and a severe combat ensued. It never occurred to the owner of the birds that the affair was anything more than a transitory love-quarrel, until the two birds fell exhausted, and both shortly died. It was found that in the combat they had stripped each other's breasts bare of feathers. So much for a family disappointment and its results.

* Dr. Laycock's Review of the Chapter in Dr. Carpenter's Human Physiology, 'On the Functions of the Nervous System," British and Foreign Medico-Chirurgical Review, No. XXIII., p. 10.

Nothing is more human than the *emulation* of song birds in the production of their song. Various examples of its fatal effects are related in books on natural history and on the instincts of animals. The singular use to which it is applied by bird fanciers amongst the lower classes, in the way of singing matches, not being generally known, is worthy of mention. These matches usually come off at some public-house or tavern consecrated to "the Fancy,"—one of these is thus described in "The British Temperance Advocate:"—

"Several members of the fraternity had brought little square bundles wrapped up in handkerchiefs; these proved to be small birdcages, each containing a pet bird. One man, opening his cage, put in his forefinger, upon which he brought out a lively goldfinch, which he offered 'to whistle again any bird in the room for a crown.' It seemed that the little songster was a celebrated prima donna in its way, and had earned the name of Jenny Lind. 'Don't you wish you may get it?' was the jeering inquiry from several voices. 'Give the long odds, and I'll match Piper again him,' bawled one; but the proposition was not accepted. The little bird plumed itself proudly, and uttered a note of defiance. 'Cock-a-doodle-doo!' screamed its proprietor; 'all afeared on yer, Jenny, that's what it is, my beauty—champion of all England, my little pinch o' feathers. Who bids ten guineas for the champion?' 'Not champion yet, if *I* know it,' said a voice from the abyss of sickening vapour; and a man stepped out of the gloom, bearing a bird perched on his knuckle, as closely resembling the redoubtable champion as it is possible to imagine. He accepted the challenge on behalf of his *protégé*, and producing his money, seated himself in a chair, rested his elbow on the table, and held forth his forefinger as a perch for the bird; the other did the same, while a third person lighted an inch of candle, and stuck it on an upturned pewter-pot between the competitors. The lists thus prepared, the challenger gave the signal by a peculiar sound, produced by drawing the air between his lips, and Jenny, after a few low and preparatory flourishes, burst into song. The rival bird responded in a strain equally loud, and both sang in evident emulation of each other, and by degrees stilled all other sounds in the room, save the snorting puffs that rose from some half hundred pipes. *The little creatures grew wondrously excited; their throats swelled, their tiny feathers ruffled up, their eyeballs rolled, their backs yawned and quivered, while, without an instant's pause or let,* amidst that horrid reek of filthy tobacco, through which their forms were but just visible, *still rushed the stream of song.* One would have thought such an atmosphere would have poisoned them, but both were plainly proof against it; and when at length the rival bird ceased, and fluttered down upon the table, it was from sheer exhaustion of physical strength, and lack of further power of endurance. Jenny, as usual, had won the day."

In comparing the working of the instincts in lower animals with the mental phenomena of man, it is easy to discover decided differences the similarities are not so much on the surface. When estimating the relations of the two classes of phenomena, it has been too much the practice to use the terms "instinct" and "reason" as antagonistic, or at least as dissimilar to a greater extent than indeed they are. So also with regard to other terms, as "voluntary" and "involuntary," and the like: we find that vague ideas and meanings are attached to them; from this vagueness ideas more vague flow, and the mind fails to perceive the true relations of things, not far severed, although apparently so. An instance of this kind is to be found in the following passage:—

"CRITES.—I may venture to make an observation which I should have made before if I had not been unwilling to interrupt the conversation. When you speak of instinct, contradistinguished to the higher faculties of the intellect, I conclude that you refer to it as a principle by which animals are impelled, independently of experience and reasoning, to the performance of certain voluntary acts which are necessary to their preservation as individuals, or the continuance of the species, or in some other way convenient to them. Now, I would ask if it be quite clear that this distinction is well-founded? Has it not been the opinion of some physiologists that, by a careful analysis of what are called instinctive actions, they may be traced to the operation of experience, quite as much as those which are palpably derived from this source?"

In this passage, animals are considered as being "impelled" to the performance of "voluntary" acts, but the terms are evidently contradictory, for that which is impelled is also compelled—a state incompatible with *voluntary* action, if by that term we mean action consequent upon an exercise of the WILL. It is, perhaps, intended to designate by the term voluntary those actions which would result if reason and will guided the animal; if that be so, it is hardly a correct use of the term. There may be, it is true, what may be termed an *instinctive* will, but then this differs widely from the rational will. How great is the difference is shown by the histories of shipwrecks, and other casualties, in which men have experienced the sharpest impulses of the instinct of hunger, to the utter discomfiture of that rational will by which, under ordinary circumstances, he is guided. In a less degree, but still obviously enough, it is manifest in the numerous instances in which man yields to temptation, and indulges his instincts and passions—a condition well known to ethical philosophers, and the source of which is tersely described by the Apostle Paul, in his own case, as "the flesh;" or, in the sententious words of Pope—in which self-love stands for all the instincts, the passions being "modes of self-love"—

"Two principles in human nature reign;
Self-love to urge, and reason to restrain;
* * * *
Self-love the spring of motion, acts the soul;
Reason's comparing balance rules the whole."

Now, the difference between instinct and reason is this, that reason acts with knowledge of the order of events, instinct without knowledge. Reason knows, and therefore adapts variously; instinct knows not, and therefore acts, according to a fixed adapting rule, blindly. Between the two extremes there are different degrees of knowledge, and, consequently, there are acts which are not wholly instinctive nor wholly rational. The "luminous exposition" which Lord Brougham has given of the mathematical accuracy with which the bee constructs its hexagonal cell, is alike a valuable example of a *pure instinct* acting *apparently*, but not really, with a profound mathematical knowledge, and of the *pure reason* which manifests that knowledge by the demonstration of the precision with which the instinct works. There are, however, an infinite variety of similar illustrations of instinctive science to be drawn from the instinctive acts of animals; as when they are

"Prescient the tides or tempests to withstand,
Build on the wave, or arch beneath the sand;
Or like the spider, parallels design,
Sure as De Moivre, without rule or line."

The perfection of this instinctive *science* is not less wondrous than its universality, for its guiding principles, as displayed in many instinctive acts, have yet to become part of our natural philosophy. We have used the term science, but it is certain that in all these there is no science in the sense of *knowledge* as there is in the analogous works of man. How or whence, then, do these instinctive powers spring? They are part of the organism, connected therewith by that intelligent, designing something in creation we have termed mind—an adapting, directing force, as inherent in living organisms as the force of gravity in matter, and like it proceeding from the Divine Mind.

Let us now accompany our author in his consideration of the human instincts. First, there are those for the immediate conservation of the organism—hunger, thirst, defence. The *social* instinct of man is thus treated:—

"Man could not exist as a solitary being. He has neither swiftness of feet, nor any natural means of offence and defence, which would enable him to compete with the buffalo, the lion, or the wolf. It would have been of little avail to him if the Creator had left it to himself to learn by hard experience that he can procure his own safety, and his means of subsistence, only by associating with others. The desire to live in society is as much an instinct in him as it is in the bee, or the ant, or the beaver, or the prairie dog. Ought not this to settle the disputed question as to the existence of a moral sense? For how could mankind live in society, helping and protecting each other, and joining in common pursuits, if they were not so constructed as to sympathise with each other in their joys and sorrows, and if they did not feel individually that they owe to others what they expect others to offer them in return? Experience and, if you please, self-interest tend to confirm, to refine, to exalt these sentiments, but they do not create them. The child is led to seek the society of other children by an impulse which he cannot resist, and which is independent of any intellectual operation. But having done so, his moral qualities, which would otherwise have remained in abeyance, are gradually developed, and (except there be some actual imperfection of the mental faculties) the power of distinguishing right from wrong, justice from injustice, follows as a matter of necessity, the result of an innate principle, and not of anything acquired."

All this is very admirable, because very true. It must not be forgotten, however, that this same doctrine is applicable to the intellectual as well as to the moral faculties. The artistic *genius*, considered as a man with *innate* powers for excellence in art, is none other than a man in whom the *instinctive* working of the mind in its special direction is nearly perfect. The illustrations of "unconscious cerebration" given by Dr. Carpenter, are drawn from a manifestation of artistic excellence which was instinctive in its possessors—Mozart, Beethoven, Coleridge. Nothing is more certain than that the poetic faculty is instinctive—*Poeta nascitur non fit.* Perhaps the instinctive working of a purely intellectual faculty is best shown in those instances in which there is the singular intuitive ability to carry on the operations of arithmetic, mentally, through a long array of figures. In all the instances we know of, the individual was hardly, if at all, conscious of the steps of the mental process; he performed it as readily as he combined any set of muscles to a given purpose; but when he proceeded to perform the calculation like ordinary mortals (and this is the strong part of the general fact), he felt it to be difficult and laborious, and was longer than most ordinary men would be. The latter method was acquired, in truth—the former was instinctive.

Let us turn now to our comparative psychology, and supply man with his instincts complete. What would he be? He would be as perfect a mathematician in his constructions as the bee; his sense of music would be perfect, of harmony of colour perfect, of form perfect; his hand would do the bidding of his instinct, and exquisite concords of sweet sounds, lustrous colouring, and perfect beauty of form would be the result. And so in every instinct manifested by lower creatures would that macrocosm—man, be complete, the most perfect of the works of the Almighty.

Now, it is not difficult to conceive an ideal man—that is to say, with intellectual and moral faculties so complete, and an organization so perfect, that he could act with all the certainty of full knowledge and all the precision of intuition—the powers of instinct and reason combined. Has man ever so existed? As to the lower instincts, the author of the "Psychological Inquiries" answers affirmatively,—as to the higher, he is silent; but leans, we think, to the doctrine of progressive development:—

"We cannot but suppose that when man first began to exist, and for some generations afterwards, the range of his instincts must have been much more extensive than it is at the present time. We see the infant first deriving nourishment from his mother's breast; but when the period of lactation is over, the experience of his parents supplies him with the fit kind of food derived from other sources. The absence of such experience must, in the first instance, have been supplied by a faculty which he does not now possess (but which we see manifested in the lower animals), directing him to seek that which is nutritious, and to avoid that which is not so, or which is actually poisonous. It is easy to conceive that much besides in the habits and actions of human beings which seem now to be the results of experience and imitation, was originally to be traced to instinct; and, indeed, there are many things which cannot well be explained otherwise. I do not venture to say that from this source he first derived the use of fire: yet it does not seem that in such an instinct there would be anything more remarkable than in that which leads the bee, with the skill of a mathematician, to construct his hexagonal cells."

These are curious speculations—more curious than useful; but it is *the* chief distinctive characteristic of man, emphatically, to be ever seeking to *know* his nature, origin, destiny. What if man were at his origin more perfect than now? What if the being now on earth be not rising to a higher than his original constitution, but rather recovering from a degradation into which he has fallen? There are facts in the natural history of man, and analogies in the histories of extinct races, which would at least cause us to hesitate in accepting that mythological doctrine of man's primæval origin and condition which has descended to modern times (unquestioned almost) through successive ages.

In the preceding considerations we have not referred to man's *religious* nature; this also has its foundation, like his intellect, in instincts. The author of the "Psychological Inquiries" advocates this view to a certain extent:—

"The disposition of man, even in his most degraded state, to believe in supernatural agencies is so universal, and so manifestly the result of his peculiar constitution, that we must regard it as having very much of the character of an instinct. As he advances in knowledge, and has leisure for observation and reflection, the perception of the beauty, grandeur, and harmony of the universe, of the evidence of intention and design, and of the adaptation of means to ends in everything around him, and of the large amount of good with the small proportion of evil which is manifested in the condition of all living creatures, leads him to the knowledge of an intelligent and beneficent Creator, to whom he *may* at any rate be responsible for the right use of the faculties with which he is endowed; and thus the religious sentiment becomes engrafted on the rude instinct of the savage."

The belief in supernatural agencies is not, it need hardly be stated, an instinct of the rude savage alone. Instances of modern belief in agencies of this kind are as common amongst the more refined and educated classes of civilized nations as amongst the rudest of semi-civilized or barbarous people. The believers in Mesmeric delusions, spirit-rapping, &c., are to be found in the highest ranks in this country.

Is the belief in those "primary and fundamental truths, the knowledge of which is forced upon us by our own constitution, and is independent of experience and reason," to be regarded as instinctive in its nature? Our author places them in a higher category:—

"It has been shown that instincts are far from being constant and immutable; as under a change of circumstances certain instincts are lost, so are others generated. Even those which are of the greatest necessity, which seem to be the most constant, may, under certain circumstances, be found to be wanting in an individual on [in] whom they had been fully developed previously. But it is otherwise with those articles of primary belief which are represented as the foundation of all our knowledge. However strange may be the illusions of the lunatic, or however convincing the arguments of the metaphysician, neither the one nor the other can escape from the belief that there is an external world independent of himself, or that what he remembers to have happened did actually occur. Taking these things into consideration, it seems not unreasonable to suppose that this class of convictions has some higher source than that which belongs to mere instincts, and that they are actually inherent in the mental principle itself, and independent of our physical organization."

To the first part of this conclusion no objection can be raised; that these beliefs are independent of organization is a proposition the truth of which is to be determined by experiment and observation alone. To our mind they show the converse to be as true of these innate sources of knowledge as of those other which are universally admitted to be dependent on physical organization. They are simply manifestations *through* organization, and *necessarily*, through organization, of what is confessedly inherent in, and indeed an essential characteristic of, the mental principle, itself distinct from, but necessarily manifested through, organization. Diseases, therefore, of the organization (as in lunacy) will and do pervert and abolish these sources of knowledge. Nothing is more a part of man's nature than the belief in his own existence; yet we have had a patient with the illusion that he was dead. If we adhere to the same definition of instinct as being part of our mental nature which is connate and innate, and by which we act to a given purpose, independently of knowledge drawn from experience, we must still, in common with most modern metaphysicians, term these beliefs *instinctive*, for they are not the less instincts because they involve what is true. On the contrary, all the instincts have this characteristic, most especially in common. Thus, when the paper-wasp makes that beautiful shelter for its young to which it owes its name, and fills it up with animal food sufficient in quantity for the *future* wants of the young being when in a higher stage of development, the instinct by which it operates is prescient of a *future* which will surely come; and this prescience necessarily implies the cosmic idea of a future. In the same way, and from the same principle, the mammæ of the human female, in common with those of all other mammalia, are prepared for the future being long before he is born. In man's mental nature this blind instinctive notion is developed into *knowledge* of the future; but in no other mode than the blind instinct of self-preservation and of abhorrence of destruction is developed into a knowledge of death, and of the means to escape it. Truth—itself inherent in instinct—is, in fact, an inherent part of man's mental nature, and, therefore, the belief in Truth. If the belief in a *future* life, so generally diffused through every tribe of man, be *instinctive* (as it is so constantly said to be), then the existence of that instinctive belief is itself a strong proof that a future life is a part of the great scheme of creation. In the same way the universal belief (instinctive, too, apparently) in the existence of God is a proof of His existence. "Quæ est enim gens, aut quod genus hominum, quod non habeat sine doctrinâ anticipationem quandam deorum?"*

The following quotation from a recent popular writer on moral philosophy (who may, indeed, claim kindred in authorship with the author of "Psychological Inquiries") is, we think, worth reprint:—"Truth being then, as I conceive, an entirely spiritual and mysterious thing, existing, like electricity, everywhere, but tangible and definable nowhere, it is vain for me to seek the discovery of it, as it relates either to things or people by any process of ratiocination; even in attempting this, I do but get into confusion worse confounded. But, observing that I possess an inner sense, quite distinct from my reason-

* Cicero, de Naturâ Deorum, Lib. i. § 16, 17.

ing powers, which in an exceedingly delicate, small, and humble way influences my apprehensions both of things and people, I wait upon this spiritual instinct as quietly and as reverently as I can; and by this sort of silent attention to its actings I gradually acquire, as I believe, a just conception of the nature of Truth."* So also Epictetus: —"It is not possible to assent to anything which appears to be not true, because it is the very nature of the understanding to agree to Truth." And Adam Sedgwick:—"Man is a religious being; * * * and though his ill-guided strivings to grasp the councils of his Maker be as powerless as the efforts of an infant in the nurse's arms to grasp the moon, still the sentiment remains an inherent part of himself; nor will all the powers of darkness root it out so long as there is a principle of causality dwelling within his soul, leading him to the conception of general truth."†

We need add no more on this head. The course of our analysis has brought us then to this conclusion—that as to his organization and the working of it, man is possessed, equally with the lower animals, of that great cosmic principle of intelligence, the self-acting *unconscious* mind. It is seen in operation throughout his entire organization. As the "nisus formativus," it outlines, completes, puts in motion the vital machinery. By it the heart is formed, as well as unceasingly pulsates during life; by it the stomach is constructed, as well as continued functionally active; by it the brain is developed, as well as put into its appropriate instinctive working; by it the entire mechanism of the organism is co-ordinated and directed towards the ends which the Creator has designed and predetermined, just as by and through it every organism acts, whether it possess a knowledge of the order of events thus designed and predetermined, or not. That is one side of the question. But superadded—plainly, surely superadded—is that other principle, the feeling, thinking, willing, self-conscious mind; the highest endowment and ultimate destiny of which seems to be a KNOWLEDGE *of the necessary order of events in creation*, of the means by which that order may be modified so as to be able to use them at will, and of the nature of the Supreme Intelligence from which all this order in creation has sprung. These two great principles meet and co-operate in organization; and the grand problem in the science of human nature is, and ever has been, to determine the relations they bear to each other therein.

We do not propose to speculate at present on this problem; the greatest minds have been bent to it in successive ages, from Plato downwards.

* Visiting my Relations, and its Results, p. 215.

† A Discourse on the Studies of the University of Cambridge. 5th Ed. Preface, p. 145.

For an admirable summary and estimate of the ancient doctrines we would refer the reader to a careful study of Cudworth's noble monument to his own genius and labours—"The Intellectual System of the Universe;" for the more modern views, the works of modern German philosophers may be consulted. The best and most recent English work is Mr. Morell's "Elements of Psychology," reviewed in a late volume of this journal.

To the medical practitioner they all have this radical defect—they are purely speculative; for what he wants as an artist and an "Ergates," is a science that can be *applied*. Such a science must be based on the relations of mind to organization, for it is wholly by communicating a knowledge of those relations that mental philosophy can be rendered available in solving all those practical questions that now embarrass him in his daily routine. What he desires to know is, the nature and treatment of insanity, the limits fixed by changes in the organization to moral responsibility, the connexion between an imperfectly or morbidly-constructed organ and crime, the best method of training and developing the mental powers, and the relations of mind to the varied pursuits of man, whatever be their nature, wherever he be, whatever be his race—the common object of all which —is HAPPINESS. In short, he desires that MENTAL PHILOSOPHY BE INDUCTIVE AND NOT SPECULATIVE.

Now an inductive mental philosophy can only be complete by including the whole range of mental phenomena within its range of inquiry. To separate the phenomena of the human mind from those which we witness in creation, would be not only to shut out from the inquiry the most fertile and most acceptable field of facts, but to take a small part only of the phenomena to be investigated, and that the most obscure. The extract from Bishop Berkley's "Siris," prefixed to the volume before us, has a most striking passage:—"*There runs a chain throughout the whole system of beings.* In this chain one link drags another; *the meanest things are connected with the highest.*" A grander passage we give from Hobbes, in his own sonorous Latin, and so clinch our argument, not by the weight of his authority, but by the force of his thought.

"Philosophiam noli credere eam esse, per quam fiunt lapides philosophici, neque illam quam ostentant codices metaphysici; sed Rationem Humanam Naturalem per omnes res creatas sedulo volitantem et de earum ordine, causis, et affectibus renuntiantem. Mentis ergo tuæ et totius mundi filia philosophia in te ipso est; nondum fortasse figurata sed Genitori mundi qualis erat in principio informi similis."*

The threefold task of the modern psychologist in summary is this—

* Ad Lectorem, in Elementa Philosophiæ. 4to. Amstelod. 1668.

1. To determine the relations of vital organization to the unconscious or kosmic reason; 2. To determine the relations of the latter to the conscious principle—the *ego* of the individual; 3. To fix the relations of the conscious and unconscious principles in combination to vital organization. The mystery of man's being will be within the reach of his intellect just in proportion as these problems are advanced towards solution. The solution can only be attained by a strict application of the inductive method to metaphysics and mental philosophy.

Something, however, has already been achieved in this direction by the inductive method. The relations of vital organization to the *unconscious* mental principle has been advanced, first, by the doctrines of reflex action, as revived and advocated by Dr. Hall; and next by the extension of those views to the cerebrum by Dr. Laycock. More recently, Dr. Carpenter has given the valued stamp of his approval to the latter, and under the term "unconscious cerebration," has considered the unconscious action of the brain in processes purely intellectual. We have elsewhere* noticed these doctrines, and refer the reader to our remarks upon them. All we need say here is this, that we believe it is no easy task to bring the mind to the conclusion that acts and processes so constantly, under ordinary circumstances, the result of the will, or at least so necessarily (apparently) accompanied by consciousness, are but automatic in their nature. Even as to the lower range of phenomena, namely, those dependent on the spinal ganglia, and which can be so easily demonstrated experimentally, this difficulty is generally felt; how much more, then, with the higher, namely, the purely cerebral and mental? The following passage may perhaps be considered as affording evidence that this difficulty has been experienced by the author of the "Psychological Inquiries," in his endeavour to keep pace with the progress of modern neurology.

"ERGATES.—It is true that Le Gallois found that certain lizards lived for a very considerable time after the loss of the head; and that when they died at last, the immediate cause of death appeared to be want. But creatures, under such circumstances, exhibit no sign of anything more than automatic life. Even breathing is suspended, the blood probably deriving the little oxygen which is required, not from air drawn into the lungs, but from being exposed to the atmosphere in the superficial vessels of the skin. It is also true that if the leg be pinched, under these circumstances the muscles are made to contract; but this is no more proof of sensibility than the starting of the limbs, which I have already mentioned as occurring in the human being in tickling the soles of the feet, after an injury of the spinal cord; or the convulsions of an epileptic patient. Then as to the multiplication of some of the lower orders of animals by division, we know so little of their

* *Vide* the Review of Dr. Noble's Lectures on the Correlation of Psychology and Physiology at p. 511.

mode of existence, and it is so entirely different from that of animals of the higher orders, that it really seems to me that we can draw from it no conclusion that would be well applicable to the latter. Is it at all certain that a polypus is endowed with any higher properties than those which belong to vegetable life? Do the motions of its filaments afford any better evidence of sensibility and volition than those exhibited by many plants, such as the *Mimosa sensitiva*, the *Dionæa muscipula*, or the *Hedysarum gyrans?* or than the folding up of many flowers in the night and in rainy weather? or than the motions of the minute bodies described under the name of cilia in animals? Or if the sensibility of the polypus be taken for granted, may it not be a compound animal, with distinct centres of sensation and volition, in like manner as in a tree every bud is, in fact, a distinct individual, which may live and grow though separated from the parent stock? An example of this mode of existence is supplied by an animal much above the polypus in the scale of living beings. The *diplozoon paradoxon* is described by Nordmann as a parasitic animal which attaches itself to the gills of the *Cyprinus Brama*. It consists, in fact, of two animals, united in the centre, so that they have a part of their viscera in common, but with two distinct nervous systems. As far as the latter are concerned there is no reason why each half of this double creature should not live very well, though separated from the other."

There is much truth in some of these remarks. It is quite certain, we think, that there has been too much assumed in investigating the class of phenomena here referred to, as to the existence or absence of feeling or consciousness. The question is one of inference and not of observation, and all experience shows that errors may be easily made either way. Thus the adaptive and conservative nature of the spinal reflex movements are so strikingly indicative of a rational will, that even yet the hypothesis—that sensation is an endowment of the spinal cord, or even of sections of it—is maintained. On the other hand, the entire absence of such movements has led observers to the erroneous conclusion that consciousness is abolished, nay, that vital action has ceased for ever. In some of these cases of apparent death (or trance-like catalepsy) the individual has possessed not only consciousness, but the sense of hearing acutely, and has comprehended every preparation for his impending interment. In the work before us we have the case of an elderly lady who recovered from a stroke of apoplexy; after the fit she lay motionless in (apparently) a state of stupor, and no one doubted that she was dying. After her recovery she explained that she did not believe that she had been unconscious during any part of the attack. She knew her situation, and heard much of what was said by those around her. The case of the late Dr. Wollaston is in various respects an interesting study. His death was occasioned by a tumour of the brain, and the history of his case proved that it must have existed from a very early period of his life. Yet perhaps that structural change rather enhanced his intellectual powers than enfeebled them, just as a blow on the head has been known to change an imbecile into a powerful mind. We subjoin the following interesting quotation :—

"During his last illness his mental faculties were perfect, so that he dictated an account of some scientific observations which would have been lost to the world otherwise. Some time before his life was finally extinguished he was seen pale, as if there were scarcely any circulation of blood going on, motionless, and to all appearance in a state of complete insensibility. Being in this condition, his friends, who were watching around him, observed some motions of the hand which was not affected by paralysis. After some time it occurred to them that he wished to have a pencil and paper, and these having been supplied, he contrived to write some figures in arithmetical progression, which, however imperfectly scrawled, were yet sufficiently legible. It was supposed that he had overheard some remarks respecting the state in which he was, and that his object was to show that he preserved his sensibility and consciousness. Something like this occurred some hours afterwards, and immediately before he died, but the scrawl of these last moments could not be deciphered."

As to vertebrate animals, it is, we think, an established fact in physiology, that consciousness ceases with the entire destruction or removal of the encephalic ganglia; so that the trunks of decapitated animals are utterly unconscious and insensible. The ordinary state of the viscera in man in relation to the consciousness, leads to the inference that in the lower articulata (if not in the higher), the consciousness hardly glimmers. But are these deductions applicable to plants? Vegetable life is so universally assumed to be, as a matter of course, unconscious, that it appears a mere folly to express a doubt of the assumption; but let a close observer and admirer of flowers watch carefully their proceedings, on the opposite assumption, namely, that they not only feel but *enjoy* life, and he will be struck with the immense array of facts which may be adduced in support of it. Endow them, hypothetically, with consciousness, and they appear to the observer in an aspect altogether different. Their instincts seem, indeed, *mutatis mutandis*, to be easily compared with those of higher animals. Unquestionably they are in the same category, in this respect, with the lower forms of *animal* life, respecting which it is impossible to determine whether they have consciousness or not.

The doctrines of reflex cerebro-spinal function have advanced our knowledge of the conscious as well as the unconscious mental principle. It is now clearly seen that there is a special arrangement corresponding to the instincts in the ganglia of the nervous system—

the centres of vesicular neurine—in virtue of which they co-ordinate and combine the various machines of the organism to fixed predetermined purposes (instinctive acts), which purposes have a tendency beneficial to the organism. That a nervous system is not *necessary* for such an arrangement in living organisms, is proved amply by the phenomena of vegetative and cell-life ; but in the higher animals it is absolutely necessary, apparently from the complexity of the machinery to be co-ordinated and combined. That special arrangement is rendered functionally active by "*impressions*" reaching the ganglia, either through the external senses or from other machines of the organism. Now if consciousness exists, it has its seat either in the ganglion, if there be only one, or in the chain of ganglia, if there be a chain, or in one or more of them set apart or "specialized" for the performance of that function; but all that we can say of consciousness itself, in its *simplest form*, is this—that when certain impressions reach the vesicular neurine, which is the seat of consciousness, the mental principle experiences a change in its condition, viz., a feeling of pleasure or of pain. If it be pleasure, then the order of events in the organism which result from the reception of the impressions are in accordance with the order pre-arranged for the good of the organism; if it be pain, then the order of events excited are inimical to the organism. Concurrently with this feeling—coincidently but not causally—there is a simultaneous action of the machinery, pre-arranged for the given end of either attaining what is good, or avoiding or repelling what is inimical.

It is not difficult to advance a stage further, and conceive another degree of consciousness; in this there is, in addition to the capability of feeling pleasure and pain, the perception that it is something external to the organism which induces the feeling—the notions of *outness* and *causation* in their simplest forms, and the foundation of the instinctive belief in the existence of an external world. This state implies the existence of a machinery for conveying impressions of external agents to the seat of consciousness, or, in other words, external senses. Still, there is neither Reason nor Will; the external agents may be desired or abhorred, according as they are excitants of pleasure or of pain, but the pre-determined arrangements in the ganglionic neurine are the source of all the *apparently* rational and voluntary *movements*.

The natural history of these pre-arranged affinities between the vesicular neurine of the periphery of the organisms and that of the central ganglia, constitutes a most important chapter in psychology. As to the intimate structure upon which they depend we know nothing further than this, that it is ultimately resolvable into cells; and the inference naturally resulting from this general fact is, that these affinities in the highest organized beings have their analogues in the cell-affinities of the simplest forms. We also know that the vesicular neurine which receives the impressions on the periphery is as specially adapted to them as the central masses. This is particularly obvious in the special senses. Whatever be the arrangements, they are constantly and necessarily transmissible from parent to offspring, for it is from them that those instincts arise and are brought into action which constitute leading characteristics in the infinite variety of animal forms. And as instincts can be acquired, so also these molecular arrangements and their dependent affinities may be acquired, and being so acquired, may in their turn be transmitted. "I walked in the fields," the author of the "Psychological Inquiries" observes, with reference to these acquired instincts, "during the autumn, with a young pointer dog, which had never been in the fields before. He stopped, and pointed at a covey of partridges." The extent to which long dormant instincts may be excited, when the appropriate impressions are transmitted through the senses, is very remarkable. The proprietors of Wombwell's menagerie sell the straw which has been used for only a few hours by their animals, when they have done with it. Such straw, that had been used by the tigers and lions, was littered in a stable as bedding for some horses. So soon as the latter entered the stable, they exhibited the greatest alarm, pricking their ears, snorting, and smelling with the utmost caution at the straw. It was evident that they detected the scent of a natural enemy, of which neither they nor their progenitors for many past generations could have been practically cognisant. The minutest habitual acts show a similar law. "In talking of hand-writing" (Moore records a conversation at Lord Denman's) "and its being sometimes hereditary, Brougham said that he had found some of his grandfather's, which exactly resembled his own, though the grandfather had died before he was born, and his father's writing was altogether different."*

There is a fundamental relation or affinity between the conscious mind and the unconscious reason, which forms and operates through these molecular arrangements. It is this: what the latter *designs* is in definite relation to the former, psychologically. Thus, the *good* of the organism is designed; now, what is good gives pleasure, and is with pleasure automatically sought after; what is inimical gives pain, and is automatically repelled with abhorrence. Again, the unconscious reason acts wholly in reference to the *external* world; the first glimmer of the conscious mind is in reference to the external world. And this idea may be evolved to a very wide extent, and include all that *knowledge* of the external world (natural philosophy) which reaches

* Memoirs, &c. of Thomas Moore, Vol. VII., p. 66.

the consciousness through the intellect, for such knowledge is possessed and acted upon by the unconscious reason to a degree far transcending man's present powers. What he may ultimately know of these cannot be fixed; it is virtually illimitable. The laws of heat, light, and electricity, so commonly applied in living organisms, have of late years had a sufficiently wonderful development to warrant the most hopeful anticipations for the future. It is in the human cerebrum that these three elements of mind have their highest development. It is a fair inference, that the vesicular organization and the unconscious reason in man are endowed, potentially at least, with as perfect powers as they display in the working of the instincts of the lower animals; it is certain that the self-conscious mind which uses them as its instruments is much more perfect. It is also a fair inference, that much of the perfection of the human mind is due to the larger surface of vesicular neurine to which it is in relation. What goes on in this vast arrangement of cells during thought is certainly at present beyond our means of research; nevertheless, if we cannot unravel the intimate nature of these physical processes, we can indicate some of their relations in varying states of the mind.* There is a state of mind, *e.g.*, in which consciousness is suspended.

"CRITES.—You have compared death from mere old age to falling asleep, never to awaken again in this world. This brings us to another subject, not very distantly related to that which we have been just discussing; at least so thought the Latin poet when he wrote—'Quid et somnus, gelidæ nisi mortis imago?' What is sleep itself? Wherefore is it required? What is the condition of the nervous system on which it immediately depends? and what, during sleep, is the actual condition of the physical and mental faculties?"

One or two general facts are stated by our author, which are of fundamental importance in the solution of these problems. All the organic processes,—the action of the heart and of the respiratory muscles, digestion, nutrition, secretion, the generation of heat—go on unceasingly; no repose is needed. Instinctive acts—indeed, all those which spring from automatic action on a fixed molecular arrangement, as the flying of migratory birds, &c.—cause no fatigue.

"The muscles of the limbs may be for a long time in a state of involuntary contraction (as in cases of tetanus or catalepsy) without weariness being induced, but under the influence of the will they cannot remain contracted for more than a few minutes at a time. In like

* There are two admirable Essays on Consciousness, in Sir Henry Holland's Chapters on Mental Physiology. The one is entitled, "Mental Consciousness, in its Relation to Time and Succession;" the other, "On Time as an Element in Mental Functions."

manner, visions may pass before the mind when it is entirely passive, without causing fatigue; but it is quite otherwise when we endeavour to arrest their progress, to view them under different aspects, and to compare them with each other. This occasions weariness, and makes us stand in need of repose, and at intervals of that complete repose which belongs to sleep, as much as voluntary muscular exertion; and these things justify the opinion, which I believe was first distinctly expressed by Dr. Darwin, that *the essential part of sleep is the suspension of volition.*"

What relations do these modes of action bear to the phenomena of insanity? The italics are our own in the paragraph just quoted; we have given emphasis to the doctrine that the essential part of sleep is the suspension of volition, because we have a deep conviction alike of its truth and that it has a direct bearing upon the relative condition of the cerebrum and the will in insanity.* But what is the will? We have seen that consciousness in its simplest form has reference to the ends designed by the unconscious mind; by parity of reasoning, the conscious will, in its simplest form as *instinctive* will, is the determination to do what the unconscious mind has designed. The perfect rational will acts from perfect *knowledge* of the ends and the means; it therefore requires sound functional activity in the organization, and a perfect co-ordination of all its parts to the end willed. The muscular system must be healthy, and in due relation to the motor part of the nervous system; the external senses must also receive impressions, and the organ of thought must duly work upon those impressions. But what happens in sound sleep? These requirements wholly fail. The muscular system (voluntary) is powerless; the motor portion of the nervous system responds either not at all or imperfectly to the will: the external senses are closed, or receive impressions imperfectly; and the organ of thought is the seat of an infinite series of changes, unconnected, uncontrolled by the will, which reach the consciousness in the forms of phantasies, illusions, dreams.

The causes of sleep are natural; that is to say, they are a part of the pre-arranged scheme of individual life. Thought implies a rest for the organ; where there is no thought—only automatic action—there is no pain nor sense of fatigue, and therefore no need of repose. The causes of insanity—of which imperfect sleep is the type—are unnatural. First and most common is work of the organ without sufficient repose. The first result of this is an inability to sleep; or, in

* The reader should especially consult Sir Henry Holland's most interesting chapter "On the Relations of Dreaming, Insanity, &c.," and the preceding one, "On Sleep," in his Chapters on Mental Physiology:—"If such phrase were permitted as a just theory of madness," Sir Henry remarks, "I know no principle so capable of affording it as that which views all the forms of insanity, including delirium, in their relation to corresponding healthy states of mind."

other words, the cerebrum continues morbidly active. The patient feels fatigued and weary, but think he must, and sleep he cannot. The following will be read with interest:—

"EUBULUS.—I have understood that this state of the system, when long continued, is sometimes the forerunner of mental derangement; and I can well understand it to be so. It is reasonable to suppose that the absence of its natural refreshment would powerfully affect the nervous system. Indeed, it happened to myself to be acquainted with a case of this kind. A gentleman of my acquaintance, in whose family circumstances had occurred which were to him a source of intense anxiety, passed six entire days and nights without sleep. At the end of this time he became affected with illusions of such a nature that it was necessary to place him in confinement. After some time, he recovered perfectly. He had never shown any signs of mental derangement before, nor had any one of his family, and he has never since been similarly affected. This was an extreme case; but do not examples of the want of sleep, producing very similar results, though in a very much less degree, occur under our observation constantly? How altered is the state of mind in any one of us after even two sleepless nights! Many a person who, under ordinary circumstances, is cheerful and unsuspicious, becomes not only irritable and peevish, but also labours under actual though transitory delusions; such, for example, as thinking that others neglect him, or affront him, who have not the smallest intention of doing either.

"ERGATES.—I have observed such effects as these repeatedly in nurses who have been harassed by an incessant attendance on sick persons during many successive days and nights; and this goes far towards explaining the origin of a vice to which individuals of this class too frequently become addicted. Alcohol removes the weary feeling and the inability of exertion which the want of sleep occasions. I have sometimes, when I have been writing late at night, and much fatigued, so that I could scarcely fix my attention on the thing before me, feeling as if my head were almost too large for the room to contain it, obtained complete relief by taking a single glass of wine."

Now, let us apply these interesting views to the pathology of insanity. Over-work, anxiety, or other depressing emotions, impure or imperfect blood, structural or functional disease of the cerebral tissues, especially from alcoholic stimuli,—all, or any of these causes of disordered cerebral action, take effect permanently upon a brain predisposed either by its original constitution or an acquired condition, to fall into the state described as resulting from want of sleep. The state is permanent —is intensified—it is insanity. The "vice" of drunkenness is now "*oinomania;*" the irritable temper is "maniacal anger;" the transitory illusions as to neglect, injury, insult, are now predominant ideas, and the affections and sentiments are perverted. The affectionate mother or gentle daughter is changed into a demon-like character, exactly the opposite to her ordinary condition. In short, we have that which is vice and crime in the healthy, appearing as "*moral insanity*" in the diseased.

The following case came under our own observation, and we can vouch for the accuracy of the details. A very worthy, pious, good man in humble life feeling indisposed, took a strong decoction of daffodils from a quack doctor. He vomited incessantly for many hours after, and then began to lose all control over his thoughts—his own expression. At the time of our visit he stated that he got no sleep, being delirious during the whole of each night; that he was extremely anxious about himself and his actions during his ravings; and in particular, that he feared that in spite of all his efforts at self-control he should be inflicting some serious, if not fatal, injury on his wife (to whom he is much attached), as during the paroxysm he feels so intense a hatred to her that he would like literally to devour her. He expressed the hope that his friends would place him in an asylum, as it was with the greatest difficulty he resisted the temptation which came into his mind to take his wife's life. We give this case as typical of a large class, which has been grouped under the term moral insanity, and with reference to the following remarks of our author.

"Crites.—It leads to another subject, in which I feel a still greater interest, partly because, from the special nature of my pursuits, it is sometimes forced upon my attention, and partly because out of it arise questions which, as they affect our social system, are of great practical importance to us all. Some writers have described, under the name of moral or instinctive insanity, a state of mind in which they say that there are no illusions, nor any affection of the intellect, but in which there is simply a perversion of the moral sentiments; the individual labouring under an impulse to perform certain extravagant and outrageous acts injurious to himself or others, such impulse being irresistible; so that he is to be held as being no more responsible for his conduct than an ordinary lunatic. Now, I own that, looking at the question merely as one who has some knowledge of human nature, and with no other aid than that of my own common sense, I am very much inclined to doubt the correctness of this doctrine, and I am certain it is dangerous to admit the plea of irresponsibility for those who labour under this so-called moral insanity, to the extent to which Dr. Prichard and others have claimed it for them. Observe that I use the term moral insanity not as comprehending cases in which there is a belief in theories that do not exist in reality, or cases of idiotcy, or those approaching to idiotcy; but limiting it strictly and exclusively to the definition given by writers on the subject. The law makes a reasonable allowance for the subsiding of passion suddenly provoked; but we are not, therefore, to presume that the same allowance is to be made for those in whom a propensity to set fire to their neighbours' houses, or commit murder, is continued for months, or weeks, or even for hours. Is it true that such persons are really so regardless of the ill consequences which may arise, so incapable of the fear of punishment, and so absolutely without the power of self-restraint, as they have been sometimes represented to be? If not, there is an end of their want of responsibility."

The author then refers to the state of the gouty patient, whose proverbial irritability is thought to be dependent upon the presence of lithic acid in the blood (though certainly bile therein is as frequent a cause), and asks whether the demonstrated presence of lithic acid ought to be admitted as an excuse for a severe bodily injury he might inflict on another in a paroxysm of ill-temper? He adds further:—

"It seems to me that juries have not unfrequently been misled by the refinements of medical witnesses, who, having adopted the theory of a purely moral insanity, ought not to be applied to at all. It is true, that the difference in the character of individuals may frequently be traced to difference in their organizations, and to different conditions as to bodily health; and that, therefore, one person has more, and another has less, difficulty in controlling his temper and regulating his conduct. But we have all our duties to perform, and one of the most important of these is, that we should strive against whatever evil tendency there may be in us, arising out of our physical constitution. Even if we admit (which I do not admit in reality) that the impulse which led Oxford to the commission of his crime was at the time irresistible, still the question remains whether, when the notion of it first haunted him, he might not have kept it under his control, and thus prevented himself from passing into that state of mind which was beyond his control afterwards. If I have been rightly informed, Oxford was himself of this opinion, as he said, when another attempt had been made to take away the life of the Queen, 'that if he himself had been hanged, this would not have happened.'"

Oxford's opinion is correct enough, no doubt, as regards *himself*, but it is hardly valid as a judgment on another.

The question of responsibility in cases of this kind is undoubtedly of vast importance, but it is also one of as vast difficulty, and only to be solved by a deep and accurate knowledge of mental physiology. We therefore do not concur in the opinion expressed by the author, "That it is a very great mistake to suppose that this is a question which can be determined only by medical practitioners; any one of plain common sense, who will give it due consideration, is competent to form an opinion on it; and it belongs fully as much to those whose office it is to administer the law, as it does to the medical profession." Common sense has erred fatally, from time immemorial, in determining the nature of all abnormal mental phenomena, for it is, in truth, only another name for popular ignorance.

We have seen that in sleep the action of the will is suspended. Now, common sense comes to our aid here, for it assures us that in dreams the most absurd and impossible notions present to our minds all the reality and verisimilitude of truths. Fortunately, the motor system cannot respond to the vagaries of the self-acting cerebrum, otherwise man would be a mischievous lunatic for a third or fourth of each day.* In delirium and in furious mania such a hypothetical state is found, for the motor system responds to the uncontrolled cerebral activity. Common sense readily judges of this mental state, for it is as obviously as abnormal as the condition of the mind in dreams. In other forms of alleged insanity, the suspension of volition is not so obvious, and therefore as to these, the question of moral responsibility resolves itself into the extent to which the power of the will is suspended. Now, as that suspension is the result and the symptom of cerebral disease, it surely follows that the men experienced in the class of diseases which suspend or destroy the will, are the proper judges and exponents of the fact of its suspension or destruction by disease. When judges are thus experienced, medical testimony will not be needed to defend the unfortunate lunatic from death or prolonged punishment; but certainly, until the bar study mental philosophy, not metaphysically, but physiologically, there must be ever the conflict between medical testimony and "common sense," for it is simply the conflict of knowledge with ignorance.

All men conversant with the insane recognise this impulsive form of insanity. They see it in different individuals, in different degrees, and in different stages. They can watch day by day the struggle between the will and the morbid impulse; now they see the one victorious, now the other, as the cerebral disorder yields or predominates. Hardly a vice can be named that is not met with in practice amongst the insane or half-insane, as a morbid impulse. A most excellent friend of ours, a man of the highest moral and intellectual culture, was seized with an impulse (at church, of all places) to commit an unnatural crime. Nothing could be more abhorrent to his nature, and happily for him, his reason told him that such an impulse could only arise in a mind diseased. He therefore fled to us for refuge, for he knew well that if the cerebral disorder attained to such a height as at once to strengthen the foul impulse and enfeeble his will, he must irrecoverably fall a victim to it. The source of the morbid condition was traced to ascarides; with their destruction, the horrid fiend vanished.

Dr. Winslow has given an exposition of this subject in his third

* "Cicero says, and justly, that if it had been so ordered by nature that we should actually do in sleep all we dream, every man would have to be bound down before going to bed :—'Majores enim, quam ulli insani, efficerent motus somniantes.'"—Sir Henry Holland.

"Lettsomian Lecture," published in the preceding number of the Psychological Journal. He there repudiates alike the term "*moral* insanity" and the disease implied. It is an assumption, a "*petitio principii*," to say that in a so-called moral insanity, *when developed into acts*, there is freedom of the will, with the power to restrain impulses and combat illusions. We do not refer to the feigned forms, but to the true cerebral disorder. In the early stages of the affection, and up to a certain point, the will may be able,—and long after the will has been put in abeyance, the intellect may *appear* unclouded (it is these circumstances, indeed, which strike the attention of the inexperienced, and constitute the difficulty of rightly discriminating between crime and disease)—but often the mental powers are really more obscured than is apparent, and very often a *sudden* increase of the morbid cerebral activity as suddenly hurls the pilot from the helm. In the lecture referred to, the question is asked, "Is the 'moral maniac' capable of pursuing an ordinary and healthy process of induction, and competent to exercise the powers of reason, comparison, and reflection, *quoad* the specific features of his so-termed 'moral' disease? He may be apparently of sound understanding; able to solve with great rapidity a difficult mathematical problem; have great capacity for the ordinary business of life; may converse with ease upon points of science, art, and philosophy; and astonish the world by the tenacity of his memory, the vividness of his fancy, the playfulness of his satire, the brilliancy of his wit, and the majesty and sublimity of his eloquence—all these elevated states of mind are compatible with *latent delusive ideas and intellectual disorder*."*

Phrenology and the "science of human nature" are the main subjects of the sixth and last dialogue in the work before us. It opens thus:—

"The term which we had allotted for our visit was drawing to a close. On the day preceding that of our departure, after wandering for some time exposed to the rays of an August sun, we found ourselves enjoying the shelter of the beech wood, which I have already mentioned as being in the neighbourhood of our friend's habitation. A tree which had been lately felled afforded us a seat. The cool shade was refreshing to us after the glare and heat of the sunshine in the open country, and the stillness and silence which prevailed afforded us the opportunity of renewing our conversation on subjects connected with those which we had discussed previously."

Those engaged in active mental labour in the busy haunts of men, universally feel—at least for awhile—how delightful is the contrast of

* Third Lettsomian Lecture, by Forbes Winslow, M.D. Journal of Psychological Medicine, July, 1854, p. 429.

sylvan retirement with town life; and the wisest almost resolve, for the remainder of their days,—

> "—— Sylvas inter reptare salubres
> Curantem quicquid dignum sapiente bonoque est."

Such feelings are rarely, however, of long duration. The "social instinct" of the citizen would soon be as irresistible as hunger, and "quicquid dignum sapiente bonoque" would be the only means of rendering a retired life endurable. The study of human nature is the highest employment of the intellect—its instincts and higher faculties, its past history, its future destiny; "in short, the 'science of human nature' taken in its most extended sense. And in this sense," adds the author, "it is a most extensive science indeed, including as it does anatomy and physiology; intellectual, moral, and political philosophy; ethnology, and I know not how much besides. Even the most abstract sciences, though not directly, are indirectly related to it, as we value them only in proportion as they tend to gratify the curiosity, supply the necessities, or elevate the character of man." With this quotation we must conclude our notice of this interesting and charming volume.

---

